# The Causation and Treatment of Sudden Dyspnoea in Goitre

**Published:** 1896-09

**Authors:** Charles A. Morton

**Affiliations:** Surgeon to the Bristol General Hospital


The Bristol

Medico-Chirurgical Journal.

SEPTEMBER, 1896.

### THE CAUSATION AND TREATMENT OF SUDDEN DYSPNŒA IN GOITRE.

### WITH REPORT OF A CASE IN WHICH THE MIDDLE LOBE OF THE THYROID WAS EXCISED FOR SUCH A CONDITION.

BY

CHARLES A. MORTON, F.R.C.S. Eng.,

*Surgeon to the Bristol General Hospital.*

A PERSON suffering from goitre, who has no dyspnœa or only a very moderate degree of it, may have a sudden and urgent attack, which may be very quickly fatal. There are various theories to account for this, but none of them seem very satisfactory. If hemorrhage has taken place into the goitre, or from the goitre into the tissues around—a condition found in a few of the cases,—we can readily understand how the sudden increase in size will produce the rapid exacerbations of dyspnœa; but such hemorrhage is very rarely found in these cases.

The theory of E. Rose[1] is founded on pathological data. He found that from the pressure of the enlarged thyroid the rings of the trachea were rendered soft and flaccid, so that after dissecting off all the other structures from the larynx and

[1] *Arch. f. klin. Chir.*, 1878, xxii., xxiii.

trachea, and then placing it upright, it gave way and bent so sharply that its lumen became completely closed. He believes that in these cases of sudden dyspnœa the trachea becomes kinked in the same way from relaxation—either during sleep or anæsthesia—of the muscles, which hold the head in such a position as to keep the tube open. But these cases do not always occur during sleep or anæsthesia, nor does relaxation of the muscles during sleep generally bring on the very urgent dyspnœa. Moreover, if it were only a question of the position of the neck, such cases would soon get relief by a change of position. They would very quickly discover they were easier by turning the neck a little more one way than another, and would relieve themselves. I have myself dissected the trachea in one case of sudden fatal dyspnœa, without discovering any softening in the rings. Mr. James Berry,[1] who has examined a very large number of specimens of goitre, has never seen this tracheal softening which Rose described; but in a case reported by Mr. C. H. Tayler,[2] the cartilaginous rings were softened and partially absorbed.

Dr. Hurry's theory of the vicious circle is very ingenious, but does not seem to me quite sufficient. He thinks that some unusual exertion may start a little dyspnœa, then that the sterno-mastoid, sterno-hyoid, and sterno-thyroid, as extraordinary muscles of respiration, come into play, and by their contraction still further press the thyroid lobes against the trachea. But these attacks are not always started by unusual exertion, and I do not think we can regard all these muscles as likely to be so active as extraordinary muscles of respiration in dyspnœa, as to seriously press a goitre against the trachea. Indeed, I doubt if the two latter could be counted as extraordinary muscles of respiration at all.

Bristowe attributed the sudden dyspnœa to inflammatory swelling and œdema of the mucous membrane. But the dyspnœa is not usually associated with symptoms of laryngeal catarrh, and there is nothing to suggest such a change in the mucous membrane.

[1] *Tr. Path. Soc. Lond.*, 1890, xli. 265. [2] *Lancet*, 1890, i. 598.
[3] *Lancet*, 1887, i. 570.

We know that although both the adductors and abductors of the cords are supplied by the recurrent laryngeal nerve, yet a lesion of the nerve may paralyse only the abductors, and hence lead to urgent dyspnœa if bilateral. Can pressure, then, on the recurrent laryngeals by the goitre be the cause of the urgent dyspnœa? The onset of the dyspnœa seems to me too sudden to be due to pressure of a goitre on the nerves. Moreover, relief cannot be obtained by opening the air-passages below the glottis, but above the goitre, as Dr. Hurry's case, before referred to, shows; and in my case two severe paroxysms came on after the laryngotomy. In the dissection of a specimen of goitre, which caused a sudden attack of fatal dyspnœa (to which reference has been made), I found one recurrent laryngeal nerve unaffected; the other had not been removed from the body before the specimen was sent to me for examination.

Patients with goitre may be going about as usual, and yet have so small a channel left for air to enter that any sudden demand for an unusual amount can scarcely be met, and hence the sudden urgency of the symptoms.[1] There is a case recorded by Jacobson[2] where sudden and fatal dyspnœa set in, apparently from fright. A woman woke up and saw her child carrying a lighted piece of wood about the room; urgent dyspnœa set in and she died. This would seem to be a case where the dyspnœa was due to some effect on the nervous system; but we must not forget that in fright the attention is fixed and the breath is held for a time, and then as the attention is relaxed the breathing is unduly rapid and full for a few seconds. Now, it seems to me that in this case the lumen of the trachea may have only just been large enough for natural breathing, and the need for increased air-entry after temporary suspension of breathing during the fright have proved too much for its capacity. In another case recorded by Jacobson,[3] where a woman with goitre died of sudden dyspnœa during labour, the explanation may be

[1] A case is recorded by Finley (*Lancet*, 1894, ii. 689) where, although only stridor was present, the trachea was found much flattened and compressed.

[2] *The Operations of Surgery*, 2nd Ed., 1891, p. 435. [3] *Ibid.*

the same: the bearing-down effort requires that the breath shall be held, and this would necessitate rapid and full breathing in the intervals, which the narrowed lumen of the trachea was perhaps unable to allow. But to support the view that the dyspnœa in these cases is due to some sudden extra demand for a larger air entry than the narrowed trachea can allow, there ought to be a history of some occurrence which usually makes people "out of breath" in all the cases; and there certainly is not, possibly because the histories are defective. In some of the cases the urgent dyspnœa came on when, so far as we know, the patient was lying quietly in bed.

A man, T. H., aged 20, came to the Bristol General Hospital under my care on May 20th, 1895, with the history that for three years there had been some enlargement of the thyroid, which had rapidly increased during the previous fortnight. All his life he had been in Bristol. He worked as a silversmith. His breathing had disturbed him at night, but there had been no attacks of urgent dyspnœa. There had not been any attack of dysphagia or giddiness. Marked tracheal stridor was present, which he said was increased by exertion, but walking up and down the ward did not increase it. There was considerable uniform enlargement of the whole thyroid. The isthmus covered the front of the trachea from the episternal notch to the cricoid cartilage, and seemed to have pushed the cricoid nearer to the thyroid cartilage than normal. The lateral lobes extended widely out under the sterno-mastoids. The right carotid was pushed out to the posterior border of the sterno-mastoid. There was no pulsation in the goitre, which felt firm. He was admitted as I considered that urgent dyspnœa might come on at any time. Ten-grain doses of iodide of potassium were prescribed, together with the local application of iodine ointment.

During the night of the 22nd there was a marked increase of the stridor, and his breathing was so laboured that he could not lie down. When I saw him at midday on the 23rd the stridor had much increased since admission; but when standing up, or lying down even when the head was raised, there was no urgent dyspnœa. I decided to do thyroidectomy on the following day. At eight p.m. that evening, when Dr. Firth—who was then House Surgeon—saw him, he was all right; but between nine and ten Dr. Firth was sent for, and found him cyanosed and unconscious. He performed laryngotomy and passed a large-sized gum elastic catheter down the trachea. The cyanosis passed off, but the breathing remained considerably obstructed. When I saw him, just before eleven o'clock, he was conscious, but not taking notice of what was going on around him. His colour and pulse were very good. On stopping up the catheter he breathed as well as before, so I removed it. I decided to excise the thyroid isthmus. Moving him up to the theatre did not increase the dyspnœa, but as soon as the administration of chloroform was commenced his breathing failed, he became of a dull leaden colour, and his pulse flagged. I got a long cannula (used for suprapubic puncture of the bladder) down the trachea, and very slowly he began to take deep, whistling inspirations through it. His colour did not improve at first, but gradually his breathing became more frequent, and then it slowly became normal. I then made an incision from the entrance of the cannula in the crico-thyroid space down to the episternal notch, and after avoiding some large veins and tying others, I reached the isthmus, which was of a dark mahogany colour, and as the fascia over it was divided, it bulged into the wound. There was a well-marked constriction where it joined the left lateral lobe. I then put two parallel silk ligatures around the junction of the isthmus and left lateral lobe, and divided the gland between them. The ligature on the isthmus slipped off, the mass of tissue being so great, and I had to catch and tie several vessels. I then ligatured the junction of the isthmus and right lateral lobe by transfixing the gland with an aneurysm needle and using two double parallel silk ligatures. The isthmus was then removed without further hemorrhage. The portion of gland excised was slightly more than one inch in width. The trachea was found to be of the typical keel-like shape, from compression of the lateral lobes, from the cricoid cartilage to well below the episternal notch. I separated it freely from the lateral lobes of the thyroid with a blunt instrument, and divided all the fascia over it. So great was the general enlargement of the gland that the cut surfaces met over the trachea, although an inch of the median portion had been excised. The hemorrhage during the operation was never serious. I irrigated the wound freely with carbolic lotion at the end of the operation, because of the presence of the laryngotomy opening, and united the edges with fishing-gut sutures, leaving a small drainage tube in the episternal notch. Once during the operation his breathing began to fail and he got very cyanosed, although he had the cannula in the trachea. I was tying some vessels in the cut surface of the isthmus at the time. All through the operation, and indeed until I left the hospital at 1 a.m., he remained quite unconscious, though his colour and pulse, with the one exception recorded, remained good. A large-sized catheter was left in the trachea. Microscopic examination of the portion of gland excised shows the enlargement to be of the ordinary parenchymatous kind.

For many days after the operation, in a quarter to half-an-hour after the removal of the catheter his voice began to fail, and he said he felt a suffocating feeling coming on, so that the catheter had to be replaced. I was able to leave it out for an hour a fortnight after the operation, and three days later it was left out for nearly a week, when the breathing became laboured and it had to be replaced for a few days longer; and then another attempt was made to do without it, but again it was required at night. It was finally dispensed with a month after the operation. Three days after the operation he got pneumonia of the lower two-thirds of the right lung; the signs of consolidation persisted for several weeks, but the continuously high temperature of 101° to 103° lasted for only three days, though it was followed by an evening rise of two or three degrees for some days longer. The thyroid was first observed to have decreased in size a fortnight after the operation, but at that time the diminution was very slight; a month later it was very marked, the left lobe being hardly palpable. Two months after operation there was very little enlargement left in either lobe, and a month later only a diffuse fulness in the thyroid region. He was able to be up three weeks after the operation, and was discharged three weeks later. The wound suppurated and healed by granulations. It was infected by the laryngeal opening. Six months after the operation he could run upstairs without any dyspnœa, and his breathing never again became laboured or stridulous. In May, 1896, there was only very slight fulness of the right lateral lobe. His voice was hoarse. Dr. Baron kindly examined the larynx and reported it normal. Possibly the hoarseness may be due to interference with the action of the crico-thyroid muscle by the cicatrix in the crico-thyroid membrane.

In this case the sudden exacerbation of dyspnœa occurred while the patient was lying in bed. After opening the air-passage above the goitre and passing a tube down the trachea it was relieved, and by the time I saw him he no longer depended on the tube for air-entry, as he breathed as easily when I plugged it. The cause of the paroxysm was no longer in operation. But a few whiffs of chloroform started the extreme dyspnœa again, and later on during the operation it started without any apparent exciting cause. The chloroform may have acted as the sudden fright did in the case I have referred to—made him hold his breath, until deeper, fuller breathing was required to compensate, of which his narrowed air-passage was incapable. But why, with the cannula in, did dyspnœa come on during the operation? The time which elapsed before the catheter could be dispensed with was very long. Possibly those degenerative changes in the rings of the trachea had taken place to which reference has already been made, and the tube tended gradually to collapse after the support of the catheter was withdrawn. The case shows that excision of the isthmus alone is not always sufficient to relieve these cases, as the catheter had to be retained for weeks after the operation.

A most instructive case bearing on this is recorded by Mr. F. Marsh, of Birmingham.[1] A girl aged 15, with a history of six months' goitre, in which the enlargement was uniform, had for several weeks marked dyspnœa, and "on one or two occasions she had almost been suffocated." The central portion, with a part of each lateral lobe, was resected. It was found that "lateral pressure had almost completely closed the lumen,

[1] *Birmingh. M. Rev.*, 1894, xxxvi. 271.

and patency had probably only been maintained by the antero-posterior counter pressure of the central part. As the pressure was removed the breathing became worse, and on completion of the excision asphyxia was imminent, the inspiratory efforts completely closing the trachea which seemed to have lost all power of expansion." Tracheotomy was performed, the largest tube available being only just sufficient. The remaining portions of the lateral lobes atrophied as the result of the excision of the isthmus. The operation was on November 20th, and the tube could not be dispensed with until the end of January, and then some dyspnœa and stridor persisted, and these did not disappear for a month later.

Another very similar case is recorded by Stevenson,[1] but the dyspnœa was greater at the time of operation. The patient was a girl, aged 16, who had suffered from goitre for two years, and dyspnœa for a fortnight. At the time of operation dyspnœa was intense, and was accompanied by cyanosis and stridor; and loss of consciousness was present for a few minutes, every now and again. The isthmus was removed, without any relief to the breathing; but after the removal of one lateral lobe as well, the dyspnœa at once greatly lessened and the girl recovered. Another case, in which the isthmus was only divided, occurred at St. Thomas's Hospital.[2] Only slight stridor was present, with a goitre of a year's duration, in a patient of the same age as the last. The isthmus was ligatured in two places and divided. Marked dyspnœa lasting several days followed the operation, and had not entirely disappeared on her discharge; and increasing again, did not subside until one lateral lobe had been excised. Berry[3] also records a case, under the care of Mr. Langton, in St. Bartholomew's Hospital, in which no relief to urgent dyspnœa in a case of goitre was obtained by division of the isthmus. In the cases operated on by Mr. Holthouse,[4] Mr. Sydney Jones,[5] Mr. Jacobson,[6] and Mr. Harsant,[7] although dyspnœa was present before operation, it was not urgent enough

[1] *Lancet*, 1891, i. 1378. [2] *St. Thomas's Hosp. Rep.*, 1894, xxii. 255.
[3] *Birmingh. M. Rev.*, 1890, xxvii. 327. [4] *Lancet*, 1875, i. 120.
[5] *Lancet*, 1883, ii. 900. [6] *Op. cit.*, p. 453.
[7] *Bristol M.-Chir. J.*, 1888, vi. 269.

to demand immediate operative interference for its relief. It disappeared after the operation, but whether as the result of the division of the isthmus or the secondary atrophy of the lateral lobes it is difficult to judge from a study of the records of the cases. Indeed, in Mr. Sydney Jones's case, one of the attacks of dyspnœa to which the patient was subject came on the day after the isthmus had been excised. It is a curious fact that the operation of excision of the isthmus was first advocated by Sir Duncan Gibb,[1] in 1875, *for the relief of dyspnœa* in bronchocele, and this is the symptom which, at any rate in urgent cases, it has again and again failed to relieve. Although from a fatal case of his own he had observed that the lumen of the trachea was reduced to a narrow fissure, obviously from pressure of the lateral lobes, yet he laid great stress on removal of the isthmus to relieve the dyspnœa. The narrowing is always lateral, from pressure of the lateral lobes—the lumen being reduced in extreme cases to an antero-posterior slit. Berry, who has examined a very large number of specimens of goitre, writes very strongly on this point. He says[2]: "I venture to formulate a rule to which I know of no exception: that the effect produced upon the shape of the trachea by a goitre of this kind namely, general uniform enlargement of the gland, is of one kind, and one only—that is, bilateral flattening." Amongst all the specimens of goitre in twenty-one pathological museums that Berry has examined, he has only discovered four cases of antero-posterior flattening, and he says that in none of these had there been any serious dyspnœa.[3] Hence it is evident that the isthmus does not itself press on the trachea so as to cause urgent dyspnœa, though a cyst formed in connection with it may do so, as in Bowlby's case, where the cyst was substernal and the trachea flattened from before backwards.[4] Does the isthmus, then, bind the enlarged lateral lobes against the sides of the trachea, and would its division or excision allow them to separate and thus relieve the dyspnœa? In my case, after excision of more than an inch of the median part of the gland, the lateral lobes came together in the middle line. Berry[5] has also observed this in one case.

[1] *Lancet*, 1875, i. 120. [2] *Tr. Path. Soc. Lond.*, 1890, xli. 265.
[3] *Birmingh. M. Rev.*, 1890, xxvii. 325. [4] *Lancet*, 1895, i. 1118.
[5] *Birmingh. M. Rev.*, 1890, xxvii. 324.

Hence it is evident that the isthmus does not hold the lateral lobes pressed against the trachea, but rather tends to keep them apart. This is, I believe, the explanation of the fact that dyspnœa is not relieved but rather increased by removal of the isthmus. What, then, should be done to relieve the urgent dyspnœa in these cases? The trachea will probably be so covered by the enlarged gland that tracheotomy below the isthmus will be impossible. A case is recorded by Tayler[1] of urgent dyspnœa in what appears to have been an ordinary goitre, in which tracheotomy was performed so low down that the aorta was felt pulsating, but the greatest difficulty was experienced in finding the trachea. A tracheotomy tube could not be introduced, and a catheter was used. The dyspnœa was relieved, but the patient died two days after of pneumonia. In another case recorded by Frost,[2] tracheotomy low down after partial division of the isthmus seems to have prevented death from an attack of urgent dyspnœa, but the goitre was of only moderate size.

Looking at the impossibility in most cases, and the enormous difficulty in others, of reaching the trachea below the isthmus, it would seem better to open the air-passage above, and pass a long cannula or catheter down the trachea past the obstruction if possible. If we could get room between the isthmus and the cricoid cartilage, so much the better; but if not, we must perform laryngotomy. We are almost certain not to have any suitable cannula at hand, and a large-sized gum elastic catheter answers quite well. The end should be cut off so as to form a terminal opening, and the stylet bent over the mouth so that it cannot protrude from the cut end and wound the trachea. I do not think we could expect to get a cannula (such as Annandale suggested) or catheter down the narrowed and perhaps deviated trachea, through the mouth and glottis, though such a method has several times relieved urgent dyspnœa when the obstruction has been at the glottis. The dyspnœa is far too rapidly fatal in many of these cases for excision of one lateral lobe to be undertaken for its relief, and excision of the isthmus alone we have seen to be useless. If the goitre is mainly

[1] *Lancet*, 1890, i. 598. [2] *Lancet*, 1890, ii. 446.

substernal, then a tracheotomy above the isthmus and an attempt to pass the catheter into the right bronchus seems the best measure we can adopt. After we have relieved the urgent dyspnœa by a laryngotomy and the passage of a catheter down the trachea, the question will arise as to the best form of excision to be undertaken. As we shall have a septic wound from the laryngotomy, we cannot expect an aseptic course in our thyroidectomy wound, and hence removal of the isthmus alone, which is a less serious operation than excision of one lateral lobe, and does not open up the structures of the side of the neck to the same degree, might with advantage be practised. Excision of the isthmus is nearly always followed by atrophy of the remaining portions of the gland.

When I performed the operation in my case, I must say I shared the general belief that it might at once relieve the dyspnœa and enable me to dispense with the catheter in the trachea; and I find it is recommended in the latest text-book of Surgery—a work published during the present year—as the best treatment for the urgent dyspnœa of goitre.

Berry has suggested[1] that division of the isthmus causes diminution in the size of the goitre by allowing the colloid secretion of the thyroid to drain away. This colloid material has been known to exude from the wound or form a collection of viscid fluid within it. But whether exudation from the cut surfaces could drain the whole goitre of colloid material seems a doubtful matter.

As urgent dyspnœa is most common in rapidly enlarging bronchoceles, when the fascia of the neck has not had time to stretch, it has been suggested that its division along the median line of the neck may give relief, the lateral lobes being thus to a certain extent freed from the compression against the trachea. Probably opening the air-passage above the goitre and passing a catheter beyond the obstruction would be more likely to succeed.

It must be remembered that compression of the trachea by a unilateral goitre always means compression by a cyst or adenoma of the thyroid, and not by ordinary goitre, and that if

[1] *Birmingh. M. Rev.*, 1890, xxvii. 327.

a cyst is present the dyspnœa might be satisfactorily relieved by its drainage if very urgent, or by its removal if less so. Urgent dyspnœa from an adenoma would require the same treatment as from ordinary goitre, but if time would allow, the adenoma could be excised.

